# Effects of Wuqinxi on joint function, muscle strength, and balance function in patients with knee osteoarthritis: a systematic review and meta-analysis

**DOI:** 10.3389/fmed.2026.1810683

**Published:** 2026-05-22

**Authors:** Shu-Ze Tang, Yu-Tong An, Yin-sheng Liang, Wen-Jie Hong, Ye Tian, Tian-Tian Zhang, Liang Ou

**Affiliations:** 1 Hunan University of Chinese Medicine, Changsha, China; 2 Affiliated Hospital of Hunan Academy of Chinese Medicine, Changsha, China

**Keywords:** meta-analysis, muscle strength, osteoarthritis, knee, postural balance, Qigong, systematic review

## Abstract

**Background:**

In recent years, an increasing number of studies have demonstrated the potential therapeutic effects of traditional Chinese exercises in the treatment of knee osteoarthritis (KOA). However, the effectiveness of Wuqinxi (WQX) for patients with KOA remains a subject of debate. This study aimed to systematically evaluate the effects of WQX on joint function, muscle strength, balance function, and pain in patients with KOA.

**Methods:**

A systematic search was conducted in seven electronic databases, including PubMed, Cochrane, EMBASE, Web of Science, CNKI, Wanfang, and VIP, from database inception to November 2025 to identify all randomized controlled trials (RCTs) on WQX for the treatment of KOA. Two reviewers independently extracted data and assessed the risk of bias using the Cochrane tool. Outcome measures included joint function scores [Lysholm and Western Ontario and McMaster Universities Osteoarthritis Index (WOMAC)], peak torque of the flexor and extensor muscles of the knee, balance function [including dynamic fall index (DFI), time to contact test (TCT), and overall stability index (OSI)], and Visual Analog Scale (VAS) scores. Subgroup, sensitivity, and publication-bias analyses were also performed. The certainty of the evidence was assessed using the Grading of Recommendations Assessment, Development and Evaluation (GRADE) approach.

**Results:**

A total of 9 trials involving 809 participants were included. The results showed that WQX significantly improved Lysholm scores [mean difference (MD) = 6.34, 95% confidence interval (CI): 2.32 to 10.36, *p* = 0.002], WOMAC scores (standardized MD (SMD) = −0.67, 95% CI: −1.08 to −0.27, *p* = 0.001), peak torque of the flexor and extensor muscles of the knee (SMD = 0.50, 95% CI: 0.36 to 0.65, *p* < 0.00001), and DFI scores (SMD = −0.95, 95% CI: −1.15 to −0.75, *p* < 0.00001). However, WQX showed no significant effects on TCT, OSI, or VAS scores. Overall, the certainty of evidence ranged from low to high.

**Conclusion:**

WQX may be an effective complementary and alternative therapy for KOA, improving joint function and muscle strength in patients with KOA. However, the available evidence is limited by geographic concentration, clinical heterogeneity, risk of bias, and low-to-moderate certainty for several outcomes. Therefore, further high-quality RCTs are needed to validate these findings.

**Systematic review registration:**

https://www.crd.york.ac.uk/PROSPERO/view/CRD420251268070, identifier (CRD420251047102).

## 1 Introduction

Knee osteoarthritis (KOA), the most common degenerative joint disorder among the elderly, is characterized by chronic pain and functional disorders (1). As a chronic progressive disease with diverse etiologies, KOA affects over 600 million people worldwide (2). With population growth and aging, its prevalence continues to rise, and the global number of patients is projected to reach 642 million by 2050 (3). Currently, there is no curative treatment for KOA, and treatment is primarily symptomatic and aims to alleviate pain (4). Common treatments include physical therapy, pharmacological approaches, rehabilitation training, and surgery (5). However, long-term medication use may lead to adverse reactions such as renal toxicity, gastrointestinal disturbances, and cardiovascular events (6).

Exercise therapy is widely recognized as a cornerstone of non-pharmacological management (7). A substantial body of research has confirmed its efficacy in alleviating pain, improving joint mobility, and delaying disease progression (8). A systematic review on exercise therapy for KOA concluded that patients can achieve significant improvements in physical function, overall quality of life, and joint pain. Exercise not only exerts positive molecular-level effects on KOA but also shows potential for symptom relief and quality-of-life enhancement, positioning it as an effective and safe non-pharmacological management strategy (9).

Traditional Chinese Medicine (TCM) emphasizes the holistic concept of “the unity of body and mind,” positing that the maintenance of life activities relies on the harmony between physical form and spirit (10). Traditional Chinese Exercise (TCE), integrating the TCM holistic view, the theories of Yin–Yang and the Five Elements, and the doctrines of meridians and viscera, has gradually developed into a unique system that combines movement and stillness, facilitates meridian flow, regulates Qi and blood, and strengthens the body for disease prevention (11, 12). Compared to general physical exercise, TCE places greater emphasis on the simultaneous regulation of body and mind. Through interventions encompassing emotional management and lifestyle adjustment, it aims to enhance immunity, harmonize Qi and blood, and improve visceral function, thereby playing an active role in disease prevention and treatment (13, 14).

In recent years, a growing number of studies have confirmed the unique value of TCE in the management of KOA (15). Tai Chi, as the most representative form, has been shown in several high-quality randomized controlled trials (RCTs) to improve pain and physical function (16). Wuqinxi (Five-Animal Exercises, WQX), an ancient Chinese health-preserving practice that advocates the principle of “the unity of form and spirit, external movement with internal stillness,” focuses not only on physical movements but also on the internal harmony of Qi and blood and mental tranquility (17, 18). Currently, the efficacy of WQX in complementary and alternative medicine remains a topic of considerable interest.

Existing meta-analyses indicate that WQX has beneficial effects on pain and joint function in KOA, but its effects on muscle strength and balance have not been fully explored (19). Therefore, we conducted a systematic review and meta-analysis to address this evidence gap.

## 2 Methods

### 2.1 Study protocol and registration

This review was reported in accordance with the Preferred Reporting Items for Systematic Reviews and Meta-Analyses (PRISMA) guidelines (20), and the protocol was registered with PROSPERO under registration number CRD420251268070.

### 2.2 Search strategy and study selection

We conducted a systematic search across seven electronic databases from their inception to 18 November 2025, including PubMed, Cochrane Library, EMBASE, Web of Science, CNKI, Wanfang Data, and VIP Database. We applied a combination of Medical Subject Headings and free-text terms related to the two concepts, KOA and WQX.

The search strategy for PubMed was as follows: ((“Osteoarthritis, Knee”[Mesh]) OR (Knee Osteoarthritides[Title/Abstract] OR Knee Osteoarthritis[Title/Abstract] OR Osteoarthritis of Knee[Title/Abstract] OR Osteoarthritis of the Knee[Title/Abstract])) AND (wuqinxi[Title/Abstract] OR “wu qin xi”[Title/Abstract] OR “wuqinxi qigong”[Title/Abstract] OR “five animal exercise”[Title/Abstract]).

Similar search strategies were used for the other databases.

The inclusion criteria were defined according to the PICOS framework:

1. Patients: adults diagnosed with KOA, accompanied by symptomatic knee pain (21–23);
2. Intervention: WQX exercise;
3. Comparison: blank control or other functional exercises;
4. Outcomes: included any of the following: Lysholm knee score (24), Western Ontario and McMaster Universities Osteoarthritis Index (WOMAC) (25), peak torque of the flexor and extensor muscles of the knee (26), dynamic fall index (DFI) (27), time to contact test (TCT), overall stability index (OSI), and the Visual Analogue Scale (VAS) for pain (28);
5. Study design: RCTs.

### 2.3 Data extraction and quality assessment

Data extraction and quality assessment were performed independently by two authors (S-ZT and Y-TA). When information regarding any of the above was unclear, we attempted to contact the trial authors for further details. Any discrepancies were resolved by a third investigator (LO).

The extracted items included: (1) author, (2) year of publication, (3) study location, (4) sample size, (5) mean age, (6) duration of KOA, (7) grade of KOA, (8) interventions, (9) controls, (10) intervention duration, (11) follow-up time, and (12) outcome measures.

The primary outcome indicators for our meta-analysis included Lysholm and WOMAC scores, and peak torque of the flexor and extensor muscles of the knee, and the secondary outcomes included DFI, TCT, OSI, and VAS scores.

The quality of the included literature was assessed using the Cochrane Risk of Bias tool (29). This tool evaluates seven domains concerning methodology, assessment, reporting, and other biases. Each domain was rated as “high risk,” “unclear risk,” or “low risk.” For some items rated as unclear, we contacted the authors to request further data for clarification.

### 2.4 Statistical analysis

Review Manager version 5.4 (Cochrane Collaboration, United Kingdom, 2020) was used to conduct all meta-analyses of the outcomes in the included studies, and the results were illustrated by forest plots.

In this review, the mean difference (MD) or standardized mean difference (SMD) was used to pool effect sizes, depending on whether the outcome measures shared the same measurement tool or unit. All pooled effects were expressed with 95% confidence intervals (CIs).

Heterogeneity was assessed using the Cochran *Q* test and the *I*^2^ statistic. An *I*^2^ value > 50% was considered to represent high heterogeneity. Subsequently, subgroup or sensitivity analyses were performed to explore sources of heterogeneity when substantial heterogeneity was present.

Random-effects or fixed-effects models were selected depending on the level of heterogeneity. Publication bias was assessed using Begg’s and Egger’s tests, which were conducted using Stata version 18. A *p*-value of <0.05 was considered statistically significant.

## 3 Results

### 3.1 Study selection

**Figure 1:**
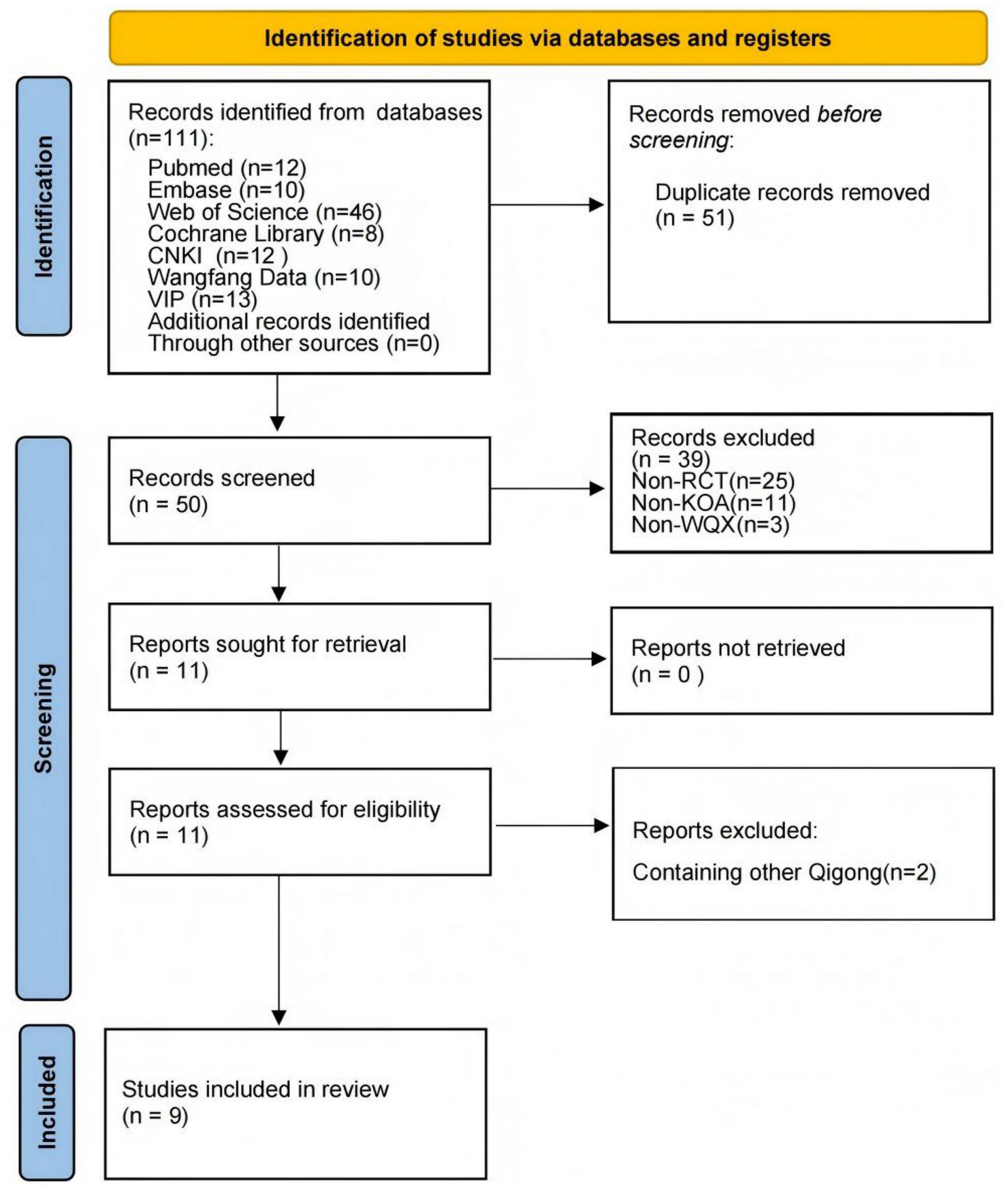
PRISMA flow diagram of the study selection process.

Through the literature search, we retrieved 111 relevant papers from 7 databases. After removing duplicates, 50 papers remained. Of these, 39 were excluded after preliminary screening because they did not include RCTs, KOA, or WQX. After further screening, two studies were excluded because their control groups involved other traditional exercises. Finally, nine studies (30–38) met the inclusion criteria. The complete screening process is shown in Figure 1.

### 3.2 Characteristics of the included studies

All studies were published within the last 14 years (2012–2025). All trials were conducted in China, and all participants were of Chinese ethnicity. The total sample size was 809, with 411 participants in the experimental group and 398 in the control group.

Most participants were diagnosed with KOA and had chronic pain lasting over 6 months and were aged over 50 years. Only seven studies reported the classification of KOA, all based on the Kellgren–Lawrence scale. Among these, four studies included participants classified as grade I/II, and three studies included participants classified as grade II/III.

All studies used WQX exercise as the intervention, with two studies also including massage (a form of Chinese physical therapy) and isokinetic muscle strength training. The control groups were either no intervention (blank control) or other forms of physical therapy. Most trials lasted between 3 and 6 months, with similar follow-up periods. The main characteristics of the extracted studies are shown in Table 1.

**Table 1:** Characteristics of data extracted from the included studies.

References	Study location	Participant characteristics				Intervention protocol				Outcome measure
		Sample size	Mean age (year)	KOA duration (months)	Grade of KOA (K–L scale)	Intervention group	Control group	Duration	Follow-up	
Li et al. (30)	Fujian, China	55/53	EG: 58.51 ± 1.20	EG: 17.64 ± 1.13	II/III	WQX + TuiNa + isokinetic training	TuiNa + isokinetic training	20 days (WQX 6 months)	6 months	A, C, D
			CG: 57.09 ± 1.22	CG: 19.26 ± 1.23						
Tian et al. (31)	Sichuan, China	20/20	EG: 63.0 ± 4.0	EG/CG ≥ 6	I/II	WQX	None	6 months	6 months	B, E, F, G
			CG: 62.0 ± 3.9							
Tu and Liao (32)	Sichuan, China	20/20	EG/CG ≥ 50	EG/CG ≥ 6	I/II	WQX	Standing exercise	16 weeks	16 weeks	B, C
Wang et al. (33)	Tianjin, China	18/10	EG: 65.00 ± 5.18	EG: 5.31 ± 4.31	II/III	WQX	None	12 weeks	12 weeks	B, C, D
			CG: 66.20 ± 5.33	CG: 5.27 ± 3.07						
Xiao et al. (34)	Beijing, China	34/34	EG: 70.7 ± 9.36	EG: 12.21 ± 7.38	I/II	WQX	Physical therapy	12 weeks	12 weeks	B
			CG: 70.2 ± 10.35	CG: 12.81 ± 5.24						
Xiao et al. (35)	Hubei, China	132/134	EG: 71 ± 2.92	EG: 28.3 ± 18.10	None	WQX	None	24 weeks	24 weeks	B, E
			CG: 69 ± 3.72	CG: 27.9 ± 17.98						
Yin and Li (36)	Anhui, China	59/59	EG: 68.6 ± 2.3	None	None	WQX	None	3 months	3 months	B, E, F, G
			CG: 69.6 ± 2.3							
Tang et al. (37)	Fujian, China	28/28	EG: 60.36 ± 4.73	EG: 19.86 ± 8.50	II/III	WQX + TuiNa + isokinetic training	TuiNa + isokinetic training	20 days	20 days	A, C, D
			CG: 59.86 ± 5 0.92	CG: 18.79 ± 8.53						
Xiao et al. (38)	Beijing, China	45/40	EG: 70.7 ± 9.36	None	I/II	WQX	Physical therapy	6 months	6 months	B
			CG: 70.2 ± 10.35							

A, Lysholm; B, Western Ontario and McMaster Universities Osteoarthritis Index (WOMAC); C, peak torque of flexor and extensor muscles; D, visual analogue scale (VAS); E, dynamic fall index (DFI); F, time to contact (TCT); G, overall stability index (OSI).

KOA, knee osteoarthritis; K–L, Kellgren–Lawrence; EG, experimental group; CG, control group; WQX, Wuqinxi.

### 3.3 Risk of bias assessment

**Figure 2:**
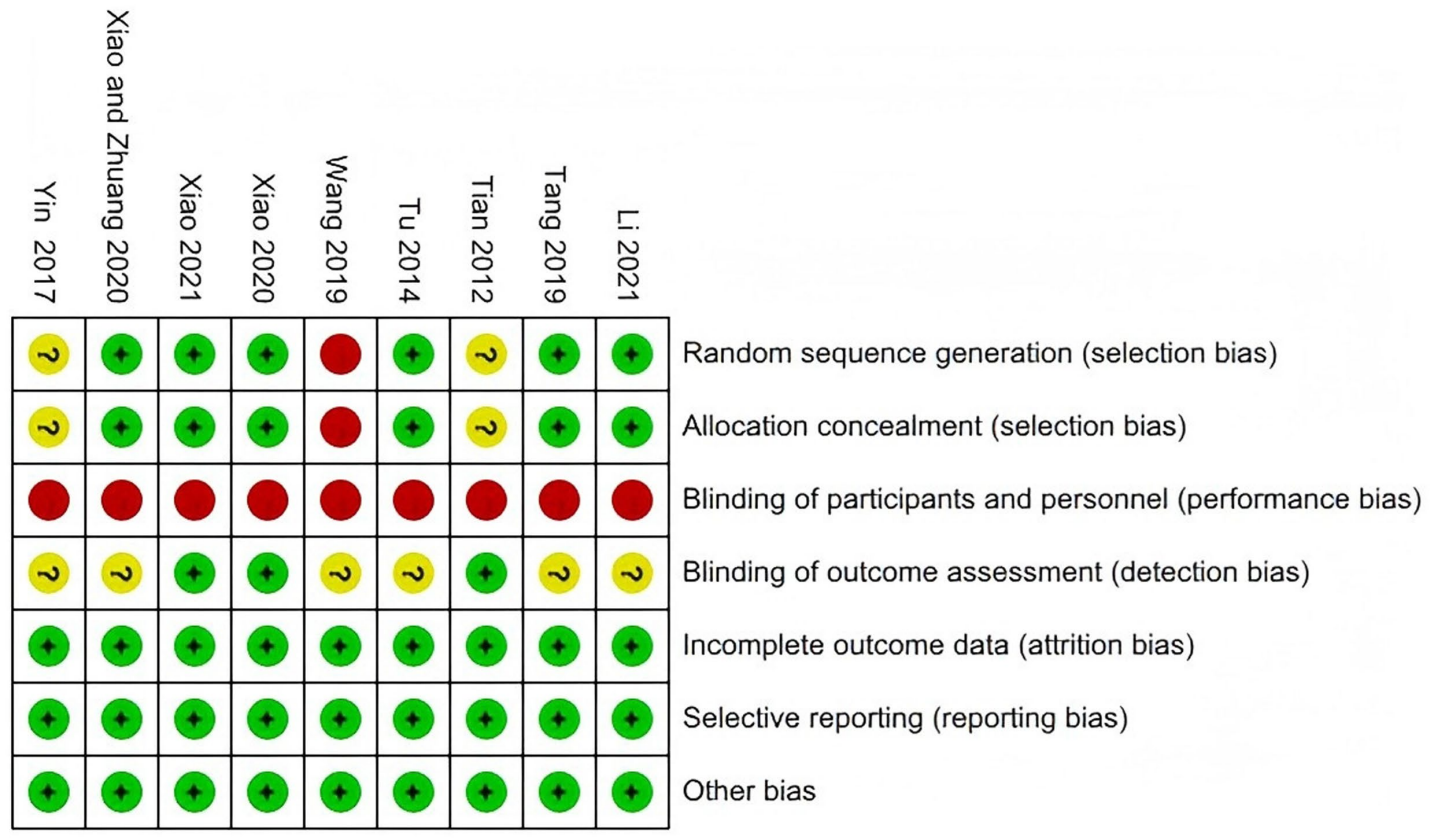
Risk-of-bias summary of the included studies assessed using the Cochrane Risk of Bias tool.

**Figure 3:**
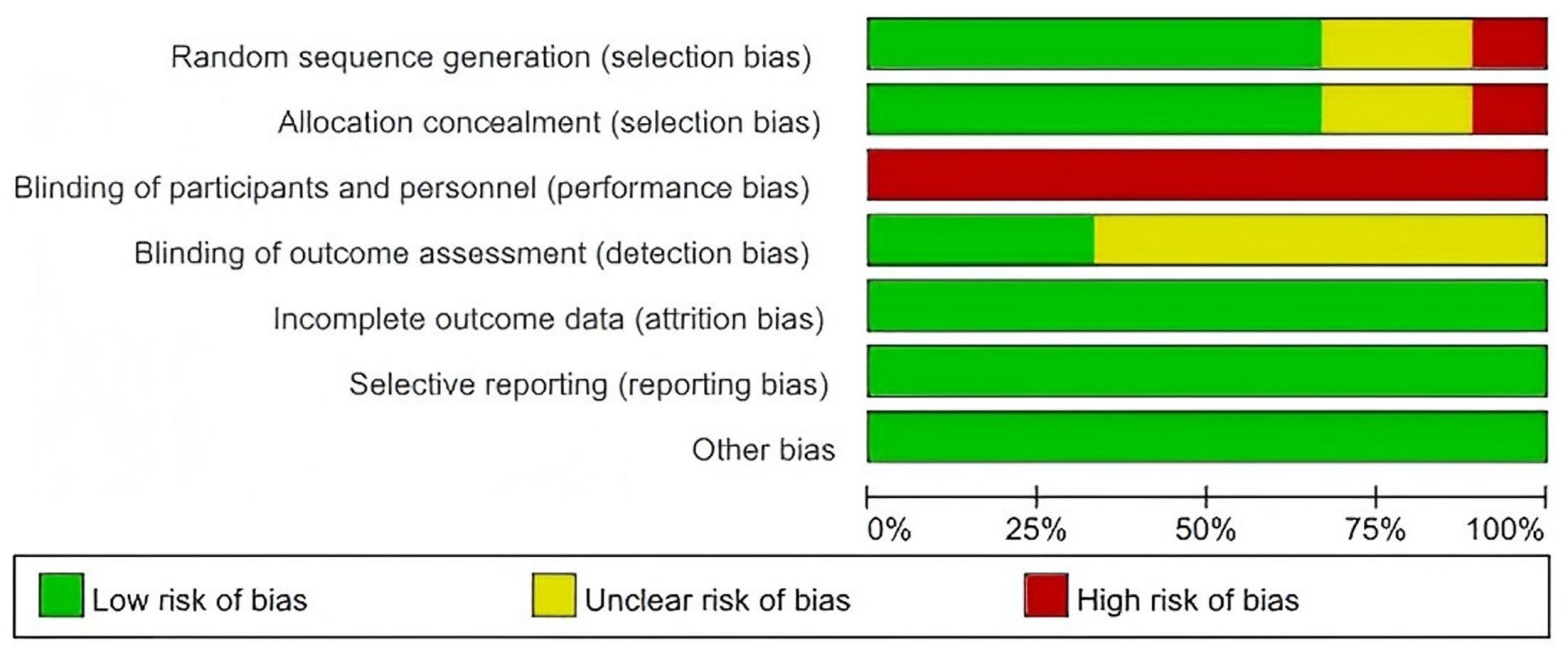
Risk-of-bias graph showing the overall distribution of judgments across the included studies.

The results are shown in Figures 2, 3. Six studies reported appropriate randomization methods and avoided selection bias. One study did not report the allocation method, and two studies did not report the methods used to generate and conceal the allocation sequence.

Regarding performance bias, all studies were judged to be at high risk because participants inevitably knew whether the experimental intervention was performed. Therefore, the overall methodological quality of the included trials was limited, reducing confidence in the pooled estimates.

### 3.4 Outcomes of meta-analysis

#### 3.4.1 Lysholm knee function scores

A total of 2 studies (30, 37) involving 140 participants were included. Both studies compared WQX plus conventional therapies with conventional therapies alone.

**Figure 4:**
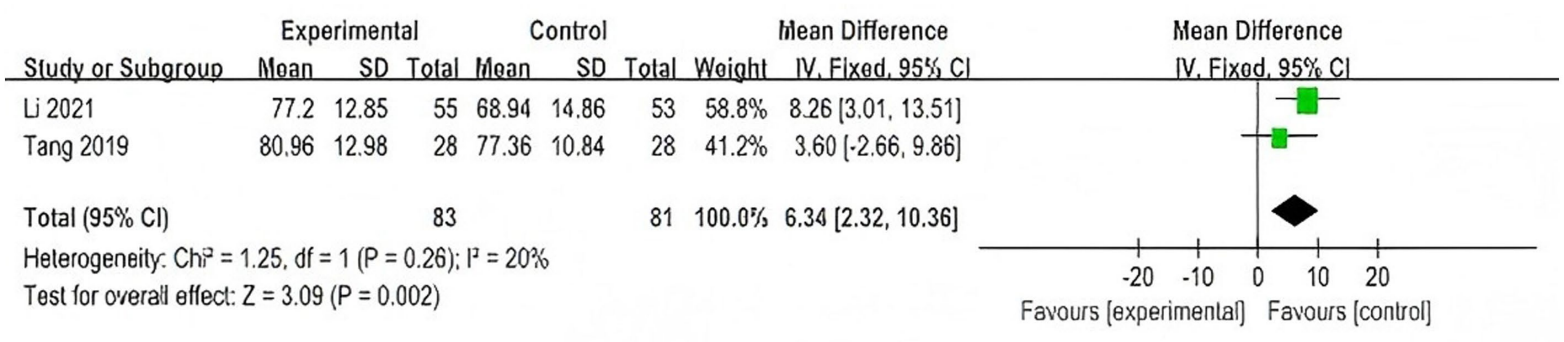
Forest plot of the effect of WQX on Lysholm knee function scores.

The meta-analysis showed that, compared with the control group, the intervention group showed improved Lysholm scores (MD = 6.34, 95% CI: 2.32 to 10.36, *p* = 0.002, *I^2^* = 20%), with low heterogeneity; therefore, a fixed-effects model was used (Figure 4).

#### 3.4.2 WOMAC scores

A total of 7 studies (31–36, 38) involving 645 participants were included. Due to differences in the versions and scoring methods of the WOMAC scores, the effect size was presented as standardized MD (SMD).

**Figure 5:**
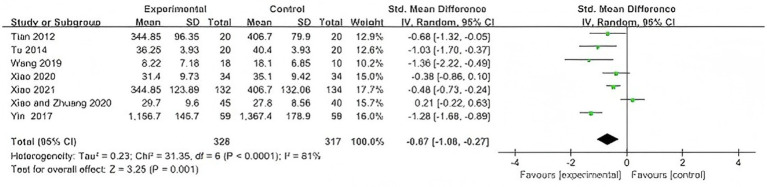
Forest plot of the effect of WQX on WOMAC scores.

The pooled results showed that WQX significantly reduced WOMAC scores compared with the control group (SMD = −0.67, 95% CI: −1.08 to −0.27, *p* = 0.001), with high heterogeneity (*I^2^* = 81%); therefore, a random-effects model was used (Figure 5).

Considering differences in control interventions (no intervention, physical therapy, and standing exercise), subgroup analysis was performed. The results showed that WQX significantly improved WOMAC scores compared with no intervention (SMD = −0.91, 95% CI: −1.40 to −0.41, *p* = 0.0004; *I^2^* = 78%) and standing exercise (SMD = −1.03, 95% CI: −1.70 to −0.37, *p* = 0.002).

**Figure 6:**
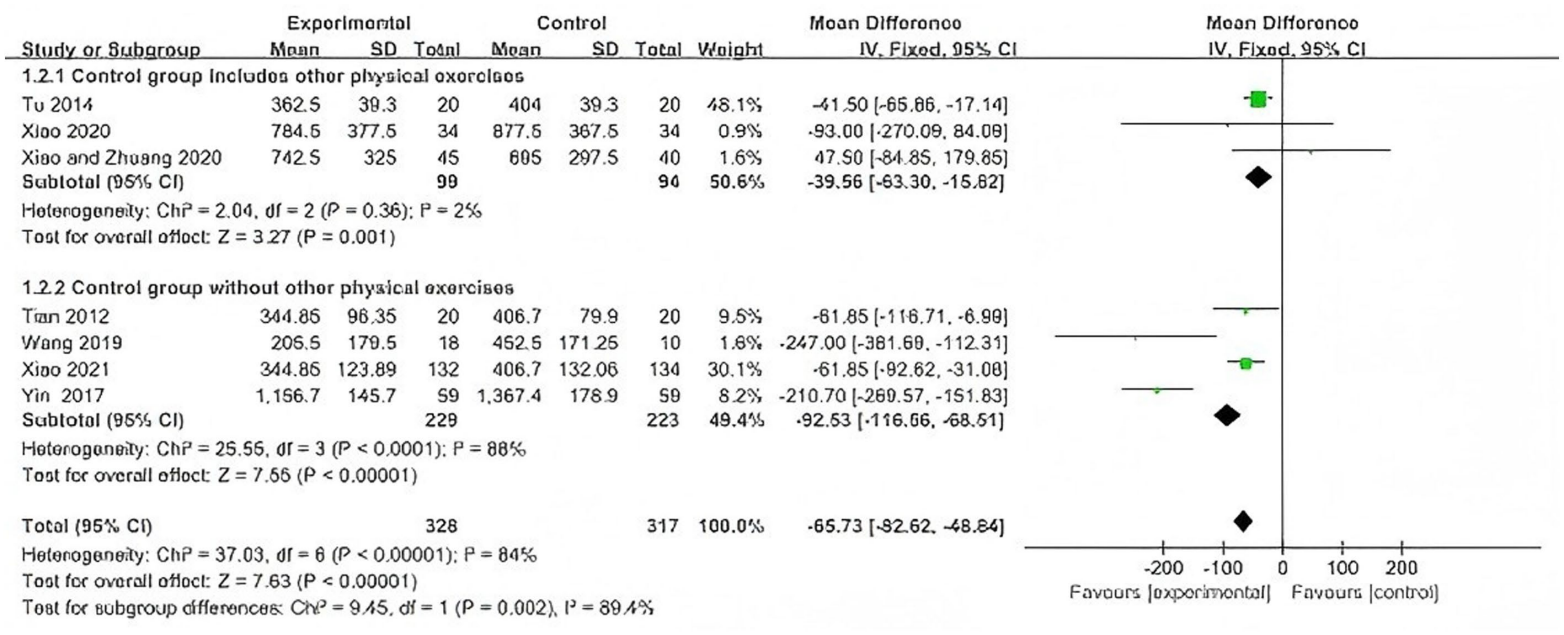
Subgroup analysis of WOMAC scores according to the type of control intervention.

However, no significant difference was observed between WQX and physical therapy (SMD = −0.08, 95% CI: −0.65 to 0.50, *p* = 0.79; *I^2^* = 69%). A borderline significant difference was observed among subgroups (*p* = 0.05) (Figure 6).

#### 3.4.3 Peak torque of flexor and extensor muscles

Four studies (30, 32, 33, 37) reported peak torque of knee flexor and extensor muscles. Considering the differences in intervention and control conditions across studies, subgroup analyses at 60°/s were conducted according to treatment design, including WQX versus no intervention, WQX versus standing exercise, and WQX plus conventional therapies versus conventional therapies alone.

At 60°/s, four studies were included (30, 32, 33, 37). The pooled results showed that both flexor and extensor peak torque were significantly higher in the WQX group than in the control group (flexors: SMD = 0.59, 95% CI: 0.32 to 0.85, *p* < 0.0001, *I^2^* = 0%; extensors: SMD = 0.42, 95% CI: 0.15 to 0.68, *p* = 0.002, *I^2^* = 76%).

Subgroup analysis showed that, for flexor peak torque, significant effects were observed in the standing exercise subgroup (*p* = 0.005) and the combined conventional therapy subgroup (*p* = 0.0005), but not in the no-intervention subgroup (*p* = 0.46), with no significant difference among subgroups (*p* = 0.41).

**Figure 7:**
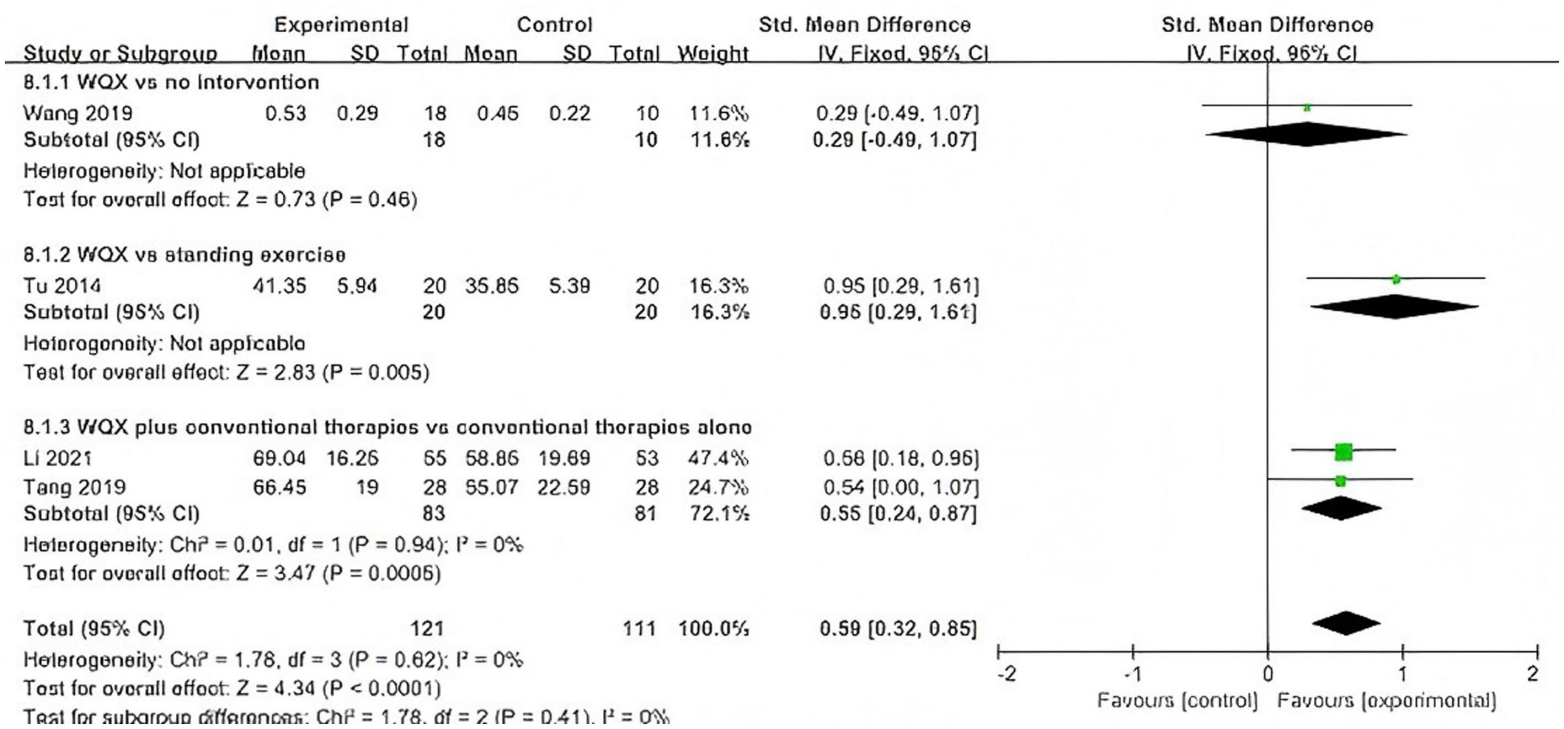
Forest plot of the effect of WQX on knee flexor peak torque at 60°/s.

**Figure 8:**
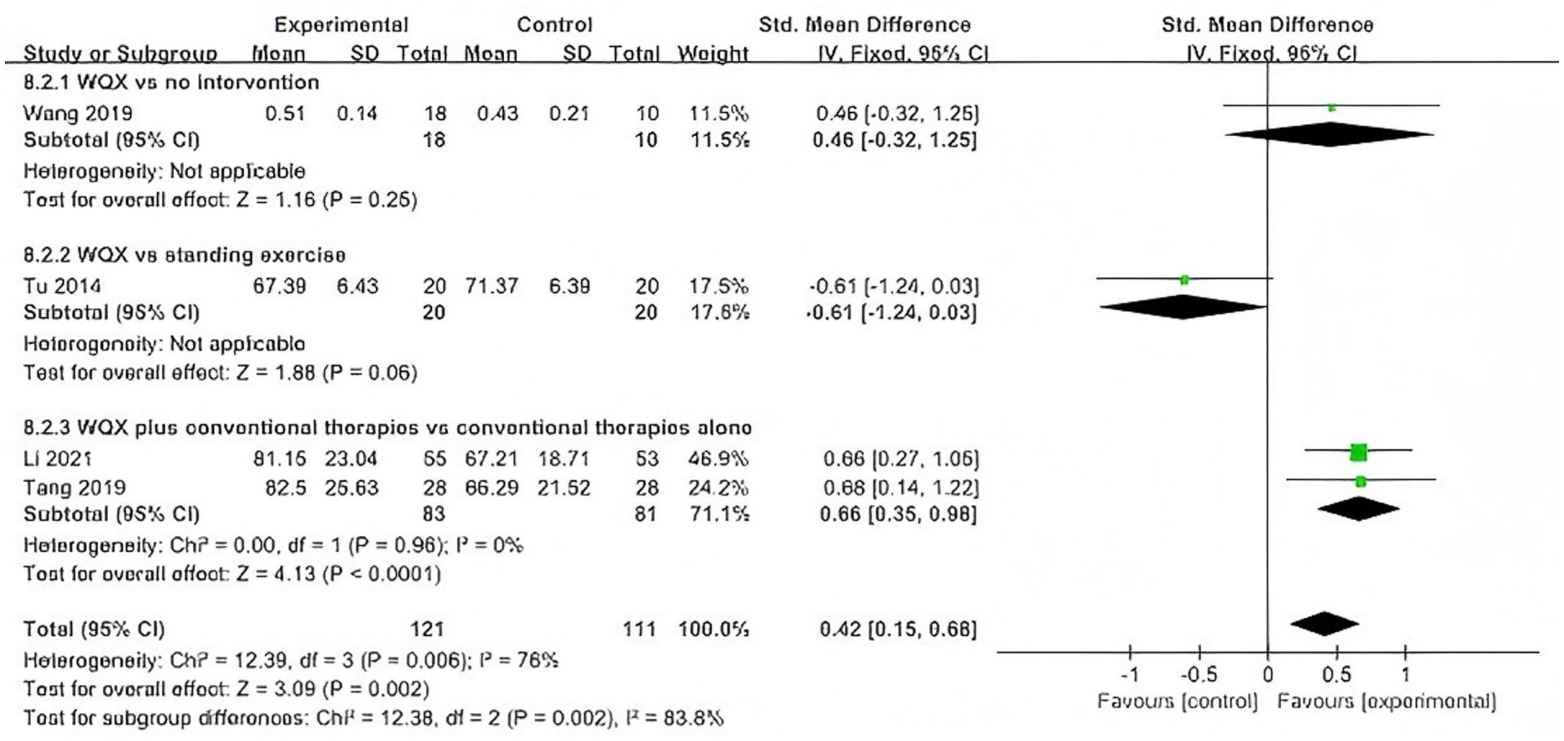
Forest plot of the effect of WQX on knee extensor peak torque at 60°/s.

For extensor peak torque, a significant effect was found only in the combined conventional therapy subgroup (*p* < 0.0001), whereas no significant effects were found in the no-intervention subgroup (*p* = 0.25) or standing exercise subgroup (*p* = 0.06); a significant difference was observed among subgroups (*p* = 0.002) (Figures 7, 8).

**Figure 9:**
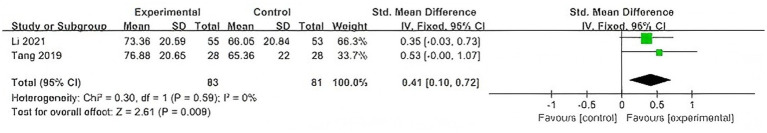
Forest plot of the effect of WQX on knee flexor peak torque at 180°/s.

**Figure 10:**
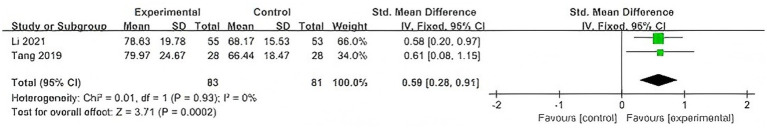
Forest plot of the effect of WQX on knee extensor peak torque at 180°/s.

At 180°/s, two studies (30, 37) were included. The pooled results showed significantly higher peak torque in the WQX group than the control group for both flexor muscles (SMD = 0.41, 95% CI: 0.10 to 0.72, *p* = 0.009, *I^2^* = 0%) and extensor muscles (SMD = 0.59, 95% CI: 0.28 to 0.91, *p* = 0.0002, *I^2^* = 0%) (Figures 9, 10).

#### 3.4.4 Balance function

##### 3.4.4.1 DFI

A total of 3 studies (31, 35, 36) involving 424 participants were included. Considering differences in DFI score interpretation, SMD was used to pool the results.

**Figure 11:**
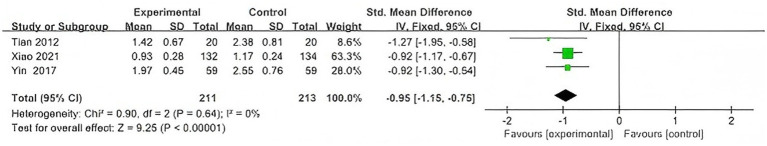
Forest plot of the effect of WQX on dynamic fall index.

The meta-analysis showed that, compared with the control group, the WQX group had better balance (SMD = −0.95, 95% CI: −1.15 to −0.75, *p* < 0.00001, *I^2^* = 0%) (Figure 11).

##### 3.4.4.2 TCT

**Figure 12:**
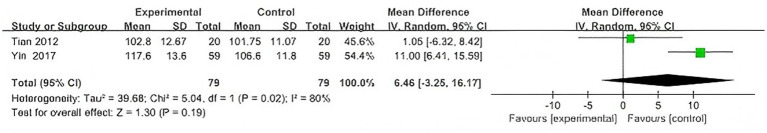
Forest plot of the effect of WQX on time to contact test.

A total of 2 studies (31, 36) involving 158 participants were included. The meta-analysis showed that, compared with the control group, the WQX group had a slight increase in TCT, but the difference was not significant (MD = 6.46, 95% CI: −3.25 to 16.17, *p* = 0.19, *I^2^* = 80%) (Figure 12).

##### 3.4.4.3 Overall stability index (OSI)

**Figure 13:**
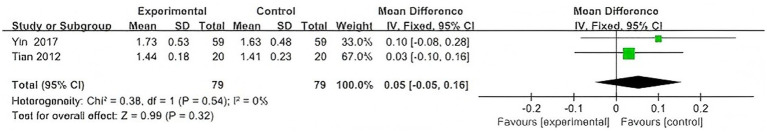
Forest plot of the effect of WQX on overall stability index.

A total of 2 studies (31, 36) involving 158 participants were included. The meta-analysis showed no significant difference in OSI between the WQX and control groups (MD = 0.05, 95% CI: −0.05 to 0.16, *p* = 0.32, *I^2^* = 0%) (Figure 13).

#### 3.4.5 VAS scores

**Figure 14:**
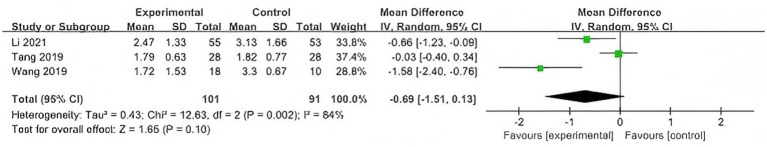
Forest plot of the effect of WQX on VAS scores.

A total of 3 studies (30, 33, 37) involving 192 participants were included. The meta-analysis showed that, compared with the control group, the WQX group reduced pain intensity, but the difference was not significant (MD = −0.69, 95% CI: −1.51 to 0.13, *p* = 0.10, *I^2^* = 84%) (Figure 14).

### 3.5 Sensitivity analysis

To verify the stability of the main results, sensitivity analyses were performed for outcomes including WOMAC scores and peak torque of the flexor and extensor muscles at 60°/s. The leave-one-out method was used to conduct the sensitivity analysis.

**Figure 15:**
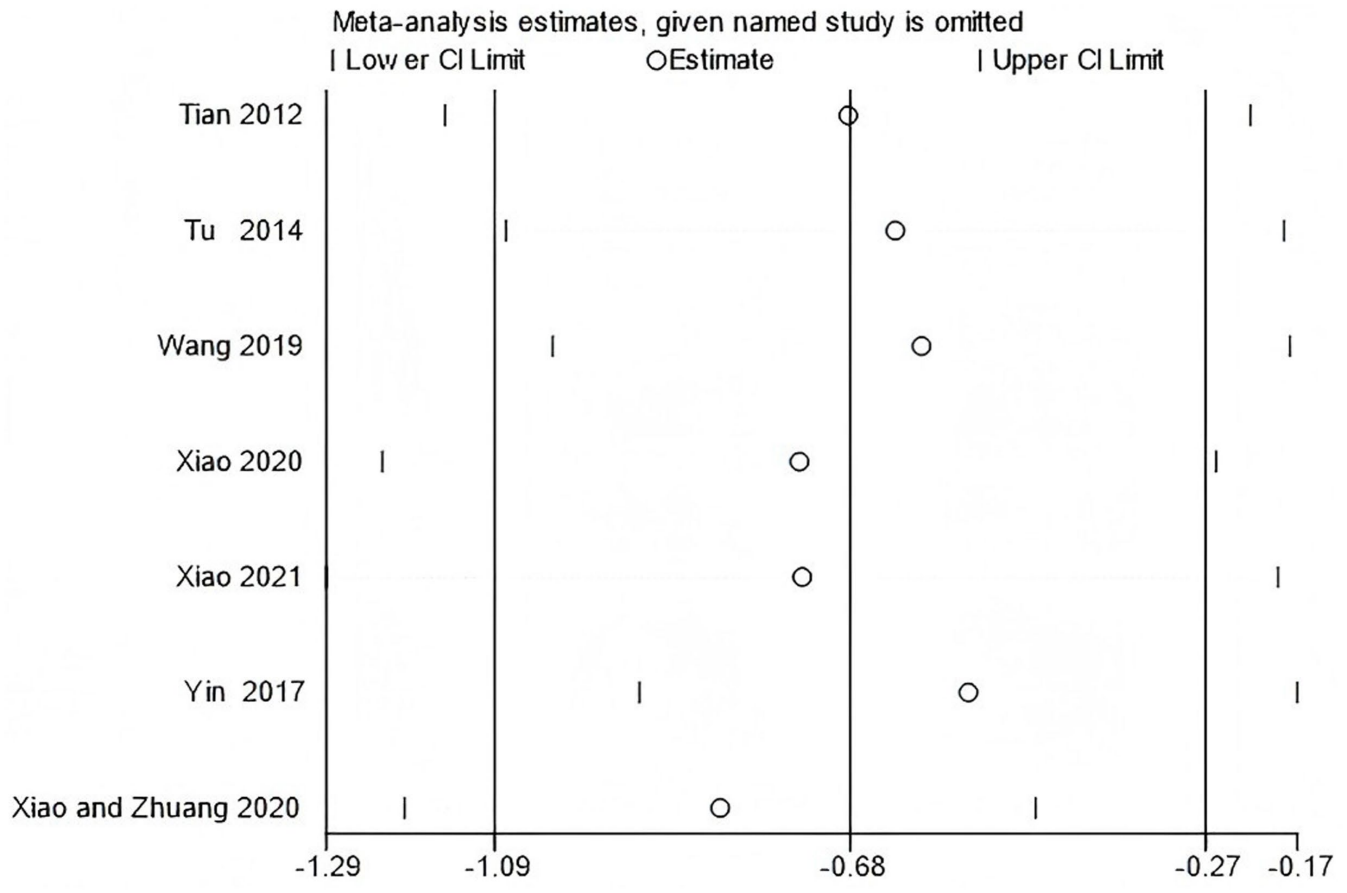
Sensitivity analysis of WOMAC scores using the leave-one-out method.

**Figure 16:**
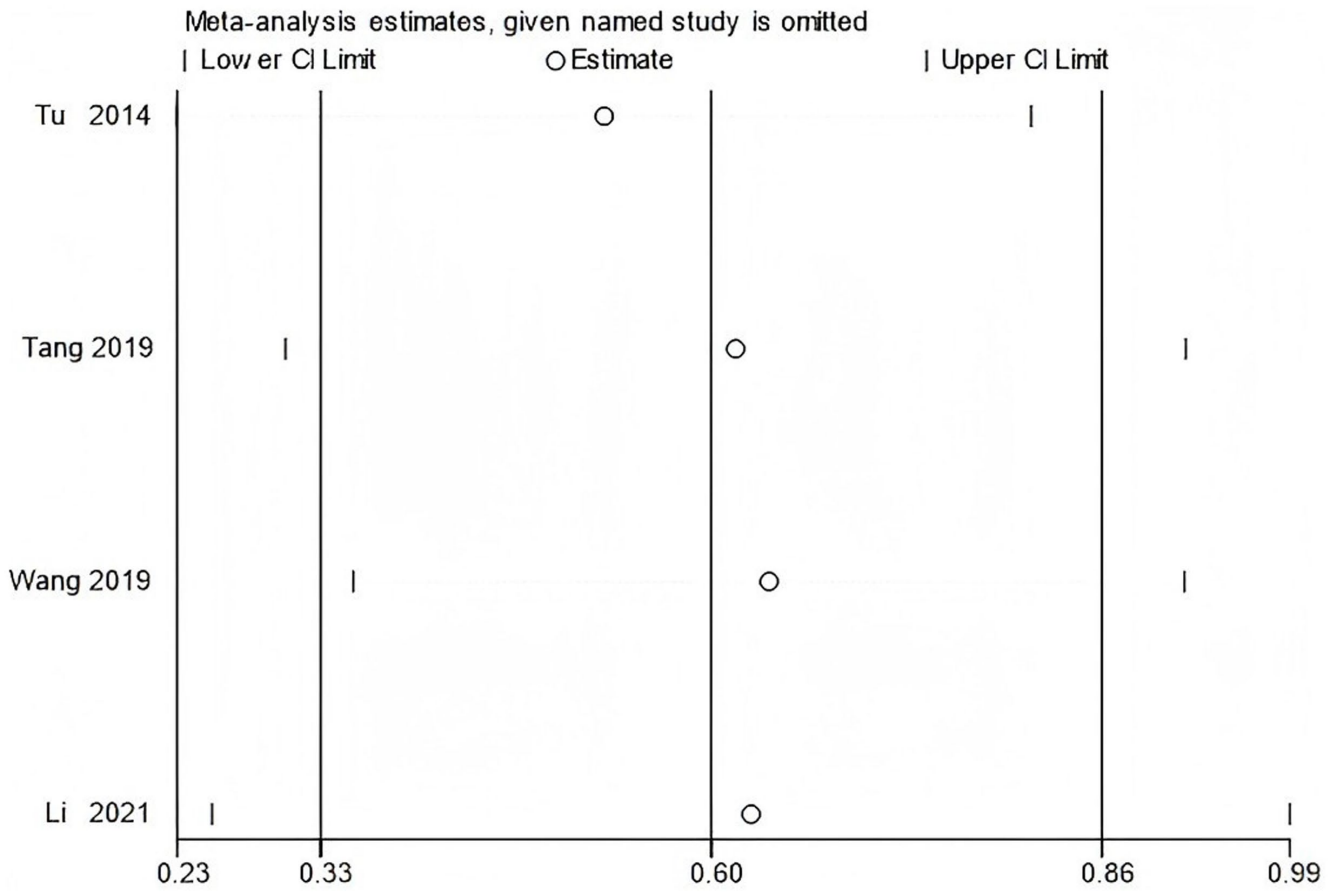
Sensitivity analysis of knee flexor peak torque at 60°/s using the leave-one-out method.

**Figure 17:**
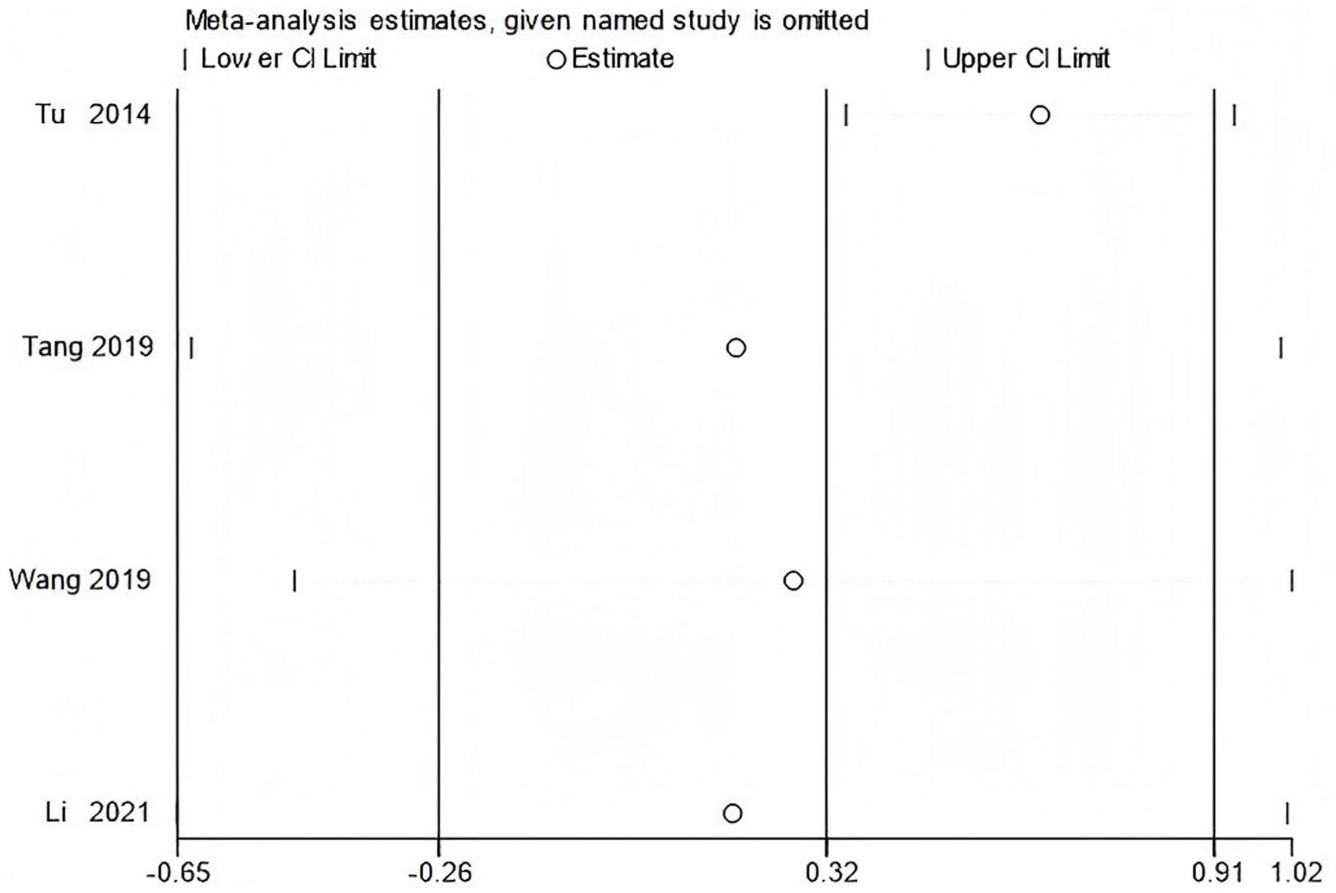
Sensitivity analysis of knee extensor peak torque at 60°/s using the leave-one-out method.

The results showed that excluding any single study did not substantially change the direction or statistical significance of the pooled effect size, indicating that the results of the meta-analysis are robust (Figures 15–17).

### 3.6 Publication bias

**Figure 18:**
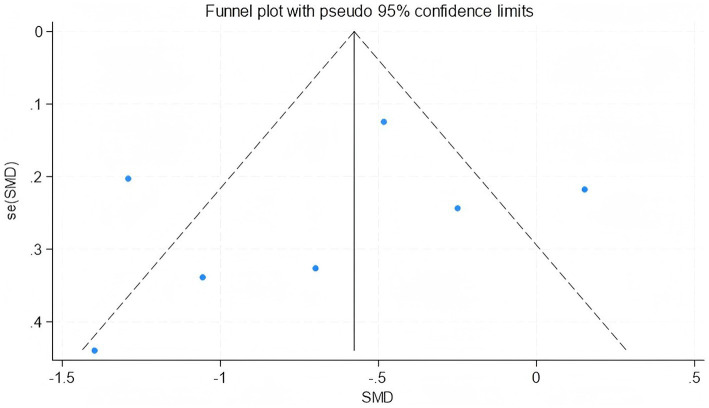
Funnel plot assessing publication bias for WOMAC scores.

Publication bias was assessed only for outcomes with more than five included studies; only WOMAC scores met this criterion. The funnel plot showed no obvious asymmetry, suggesting a low risk of publication bias (Figure 18).

**Figure 19:**
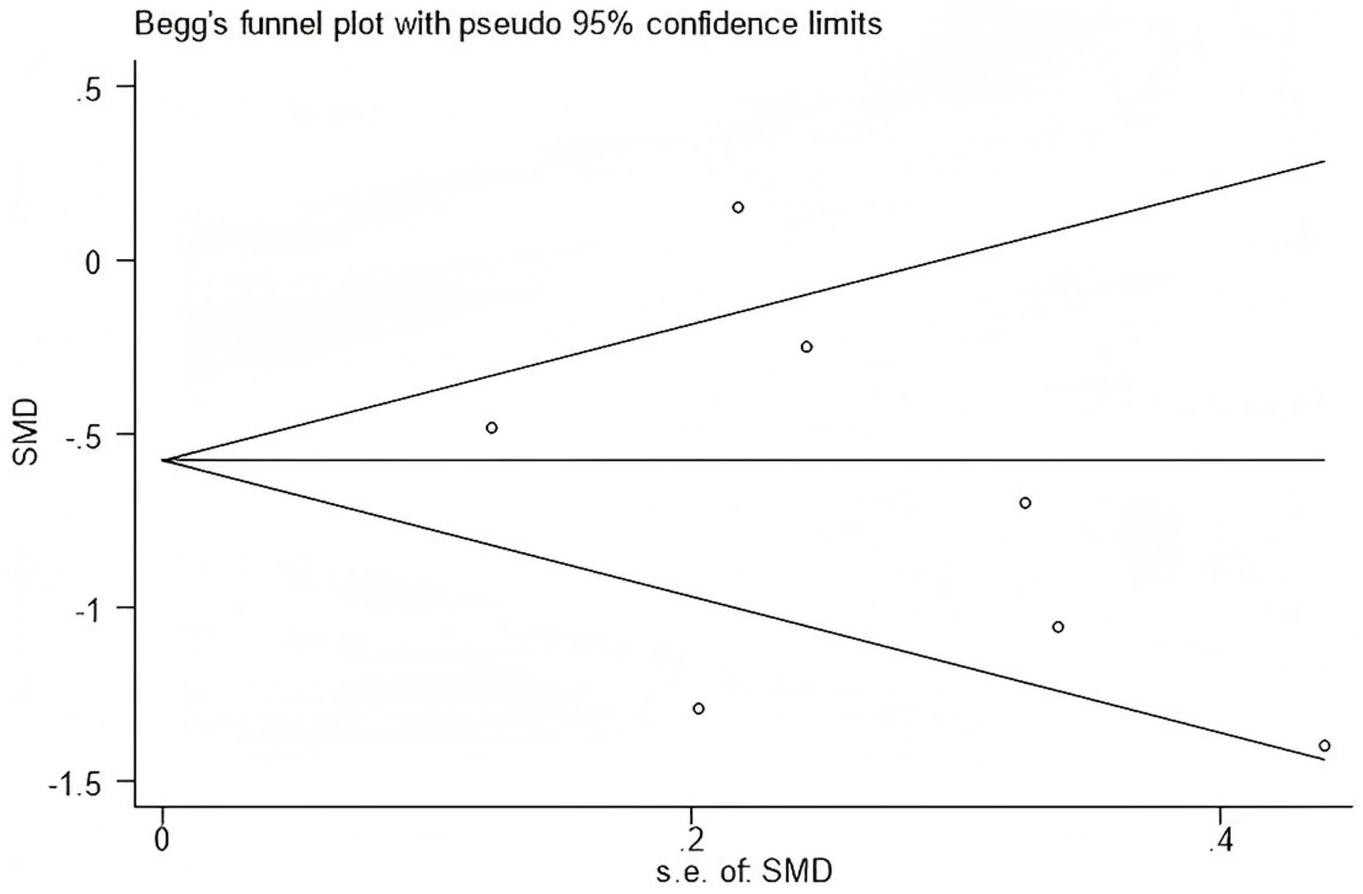
Begg’s funnel plot assessing publication bias for WOMAC scores.

**Figure 20:**
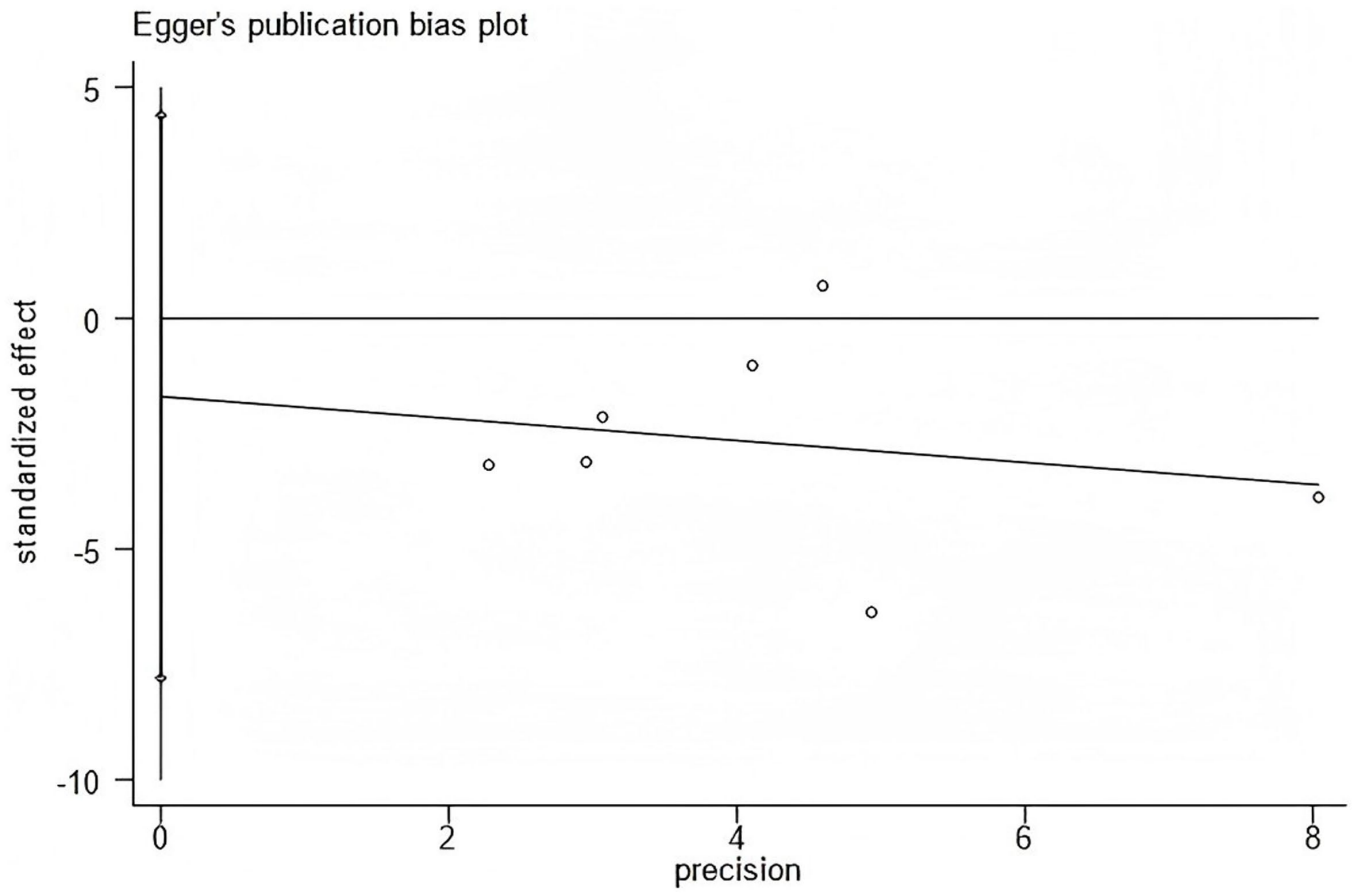
Egger’s publication bias plot for WOMAC scores.

Furthermore, Begg’s and Egger’s tests did not identify significant publication bias (Egger’s test, *p* = 0.662; Begg’s test, *p* = 0.293) (Figures 19, 20).

### 3.7 Evidence quality assessment

The quality of evidence was assessed using the Grading of Recommendations, Assessment, Development, and Evaluation (GRADE) system.

The summary of findings indicated low certainty for WOMAC and VAS scores, moderate certainty for peak torque outcomes, and high certainty for Lysholm, DFI, TCT, and OSI (Table 2).

**Table 2:** GRADE assessment of the certainty of evidence for all outcomes.

Variable	No. of studies	No. of participants	Effect estimate (95% CI)	*I^2^* heterogeneity, %	GRADE
Lysholm	2	140	6.34 (2.32 to 10.36)	20	High
WOMAC scores	7	645	−0.67 (−1.08 to −0.27)	81	Low
Peak torque					
Flexion peak torque (60°/s)	4	232	0.59 (0.32 to 0.85)	0	Moderate
Flexion peak torque (180°/s)	2	164	0.41 (0.1 to 0.72)	0	High
Extension peak torque (60°/s)	4	232	0.42 (0.15 to 0.68)	76	Moderate
Extension peak torque (180°/s)	2	164	0.59 (0.28 to 0.91)	0	High
DFI	3	424	−0.95 (−1.15 to −0.75)	0	High
TCT	2	158	6.46 (−3.25 to 16.17)	80	High
OSI	2	158	0.05 (−0.05 to 0.16)	0	High
VAS scores	3	192	−0.69 (−1.51 to 0.13)	84	Low

CI, confidence interval; *I*^2^, inconsistency statistic; WOMAC, Western Ontario and McMaster Universities Osteoarthritis Index; DFI, dynamic fall index; TCT, time to contact test; OSI, overall stability index; VAS, visual analogue scale.

## 4 Discussion

### 4.1 Joint function

Exercise therapy, as a supplementary and adjunctive physical treatment, can prevent cartilage degeneration, suppress inflammation, and prevent the loss of subchondral bone and trabecular bone (39). Studies have shown that exercise therapy can increase muscle cross-sectional area, reduce muscle fiber density, alter tendon structure, and delay musculoskeletal atrophy, thereby improving joint stability, reducing inflammation, improving synovial cell function, and preventing cartilage degeneration and subchondral bone loss (40). Therefore, exercise therapy plays a crucial role in improving knee joint function in patients with KOA.

Pooled results from two studies demonstrated that WQX, when added to conventional therapies, improved Lysholm scores with low heterogeneity, suggesting a potential adjunctive benefit on knee function. However, this result reflects the additional effect of WQX on top of conventional treatment, rather than its isolated effect.

For WOMAC, seven studies suggested an overall benefit of WQX, but substantial heterogeneity remained. In the subgroup analysis, the benefit was more evident when WQX was compared with no intervention or standing exercise, whereas no significant difference was found when compared with physical therapy. This suggests that the comparator type may partly explain the heterogeneity. In addition, differences in exercise frequency, intervention duration, and home-based adherence may also have contributed to variability in WOMAC outcomes. Therefore, the WOMAC findings should be interpreted cautiously.

### 4.2 Muscle strength

Many studies, both domestic and international, recognize that muscle strength plays an important role in the onset and progression of KOA (41). Scholars worldwide agree that decreased knee muscle strength has many negative effects on the daily life of patients with KOA (42, 43).

Knee extensor muscles are essential for joint stabilization and shock absorption during gait, and reduced extensor strength may compromise tibial control, increasing the risk of structural knee damage and contributing to disease progression. Although knee flexors are generally considered less influential than extensors in osteoarthritis progression, emerging evidence suggests that reduced flexor strength is associated with an increased risk of tibiofemoral osteoarthritis deterioration, highlighting the importance of flexor function for joint health in patients with KOA and at-risk populations (44).

The present meta-analysis demonstrated that WQX was associated with improved peak torque of both knee flexor and extensor muscles in patients with KOA. At 180°/s, the pooled effects for both flexor and extensor peak torque were significant, with no observed heterogeneity, indicating a relatively consistent benefit under this testing condition.

At 60°/s, the pooled results also favored WQX for both flexor and extensor strength. However, the included studies differed in treatment design. Some studies evaluated WQX as a standalone intervention, whereas others assessed its additional effect on top of conventional therapies.

In the subgroup analysis, no significant subgroup difference was observed for flexor peak torque, suggesting that the beneficial effect on flexor strength was relatively stable across different comparison settings. In contrast, a significant subgroup difference was observed in extensor peak torque, indicating that the treatment design may have contributed to the heterogeneity in the extensor analysis.

This interpretation is supported by the included trials: WQX showed greater improvement than standing exercise in one study, whereas in the trial using physical therapy as an active comparator, between-group differences in knee flexor and extensor strength were not significant.

Therefore, WQX may improve knee muscle strength in patients with KOA, but the magnitude of benefit may vary depending on the comparator type and whether WQX is used alone or as an adjunct to conventional therapy.

### 4.3 Balance function

Balance refers to the ability of a system to control its center of gravity within a given environment (static or dynamic), thereby maintaining posture without falling (45). The quality of balance directly affects the safety of daily activities and mobility in patients with KOA (46).

This meta-analysis explored the effects of WQX on balance using three indicators: DFI, TCT, and OSI. The pooled results demonstrated that WQX significantly reduced DFI, with no observed heterogeneity across studies. This finding suggests that WQX may enhance dynamic balance control in patients with KOA.

The slow, coordinated, and continuous movement patterns characteristic of WQX may improve proprioceptive input and neuromuscular regulation, thereby facilitating postural adjustments during movement and reducing fall risk, which is clinically relevant for rehabilitation.

In contrast, no statistically significant improvements were observed for TCT or OSI. The pooled estimate for TCT showed substantial heterogeneity, whereas OSI showed low heterogeneity but no significant effect.

The heterogeneity in TCT may be related to differences in intervention duration, study setting, participant characteristics, and testing procedures, even though both included studies used no-exercise controls. Therefore, while WQX appears to reduce dynamic fall risk, its effects on TCT and OSI remain inconclusive.

### 4.4 VAS scores

For VAS scores, this study found that the pooled effect did not reach statistical significance, which differs from previous studies. The evaluation of the analgesic effect of WQX should therefore be interpreted cautiously.

The study by Guo et al. (19) reported statistically significant improvements in VAS scores, whereas the present analysis did not. This discrepancy may be due to the inclusion of additional studies with different effect sizes [such as Tang (37)], which may have influenced the pooled results.

Differences in treatment duration, follow-up design, and exercise adherence may also have contributed to heterogeneity. Therefore, the analgesic effect of WQX should be interpreted cautiously.

Several limitations should be noted. First, all included studies were conducted in China, which limits the generalizability of the findings. Second, there was substantial clinical heterogeneity across studies in terms of intervention format (WQX alone versus WQX combined with other treatments).

Third, several outcomes, including WOMAC, VAS, and TCT, showed substantial statistical heterogeneity, and the small number of studies for some outcomes limited further exploration of heterogeneity sources.

Fourth, the methodological quality of the included trials was limited, particularly with respect to randomization reporting and allocation concealment.

Finally, adverse events were not systematically reported; therefore, the safety of WQX could not be reliably assessed in this review.

## 5 Conclusion

This systematic review and meta-analysis suggest that WQX may provide benefits for joint function, muscle strength, and balance function in patients with KOA.

However, the current evidence is limited by geographic concentration, clinical heterogeneity, and methodological limitations. The findings should be regarded as preliminary rather than definitive.

More rigorous and internationally representative RCTs are still needed before firm clinical conclusions can be drawn.

## Data Availability

The original contributions presented in the study are included in the article/supplementary material, further inquiries can be directed to the corresponding authors.
